# Clonal Diversity, Antibiotic Resistance, and Virulence Factor Prevalence of Community Associated *Staphylococcus aureus* in Southeastern Virginia

**DOI:** 10.3390/pathogens13010025

**Published:** 2023-12-27

**Authors:** Katelyn D. Cranmer, Mohan D. Pant, Suzanne Quesnel, Julia A. Sharp

**Affiliations:** 1 Microbiology and Molecular Cell Biology, Eastern Virginia Medical School, Norfolk, VA 23507, USA; 2 School of Health Professions, Eastern Virginia Medical School, Norfolk, VA 23507, USA; 3 Children’s Hospital of the King’s Daughters, Norfolk, VA 23507, USA

**Keywords:** virulence factor, immune evasion, antibiogram, community associated, complement evasion, infection, MRSA, drug resistance, whole-genome sequencing

## Abstract

*Staphylococcus aureus* is a significant human pathogen with a formidable propensity for antibiotic resistance. Worldwide, it is the leading cause of skin and soft tissue infections (SSTI), septic arthritis, osteomyelitis, and infective endocarditis originating from both community- and healthcare-associated settings. Although often grouped by methicillin resistance, both methicillin-resistant (MRSA) and methicillin-sensitive (MSSA) strains are known to cause significant pathologies and injuries. Virulence factors and growing resistance to antibiotics play major roles in the pathogenicity of community-associated strains. In our study, we examined the genetic variability and acquired antibiograms of 122 *S. aureus* clinical isolates from SSTI, blood, and urinary tract infections originating from pediatric patients within the southeast region of Virginia, USA. We identified a suite of clinically relevant virulence factors and evaluated their prevalence within these isolates. Five genes (*clfA*, *spA*, *sbi*, *scpA*, and *vwb*) with immune-evasive functions were identified in all isolates. MRSA isolates had a greater propensity to be resistant to more antibiotics as well as significantly more likely to carry several virulence factors compared to MSSA strains. Further, the carriage of various genes was found to vary significantly based on the infection type (SSTI, blood, urine).

## 1. Introduction

*Staphylococcus aureus* causes a multitude of infections that affect various bodily systems, including the skin and soft structure, bone, joints, heart, bacteremia, and implant- or surgical site-affiliated infections [1,2]. Whilst healthcare-associated (HA) infections are often the focus of preventative measures imposed by care facilities and hospitals, community-associated (CA) infections, which originate outside of a healthcare setting, remain a significant contributor to the overall burden of *S. aureus* on healthcare systems worldwide [3,4,5,6]. Both its persistence within environmental and animal reservoirs [2,7,8], as well as a high rate of benign carriage within most healthy populations (about 30%) [2,9], contribute to this ongoing threat to community health. In recent years, the rate of infection by methicillin resistant *S. aureus* (MRSA) has decreased, stabilizing increases seen in the late 1990s to early 2000s [4,10], with a shift in concern to vancomycin and clindamycin resistance currently [11,12,13,14,15]. MRSA infections remain a significant source of disease worldwide, particularly within vulnerable populations. Risk factors include previous hospital stays, catheterization, advanced age or infancy, intravenous drug use, being of an ethnic minority, or lower socioeconomic status [3,4,5,6,16].

*S. aureus* wields a plethora of virulence factors (VFs) designed to facilitate infection and cause disease. To survive within the host environment, *S. aureus* adheres to host surfaces, utilizes host elements for metabolic needs, and subverts host immunity through a variety of VFs. The complement system, operating through a catalytic cascade, plays a significant role in the host innate defense by labeling pathogens as foreign (opsonization), lysing susceptible cells, and actively recruiting effector cells for pathogen clearance. As such, the complement system and its various components are primary targets of *S. aureus* VFs [17].

Adhesins, which allow *S. aureus* to bind to a host substrate and promote biofilm development, can be subdivided into cell wall-bound MSCRAMMs (microbial surface components recognizing adhesive matrix molecules) and secreted SERAMs (secretable expanded repertoire adhesive molecules). MSCRAMMs have at least two IgG-like folds and a “dock, lock, and latch” mechanism for binding to ligands (reviewed in [18]). SERAMs mediate bacterial adhesion with host components (such as cells, molecules, or tissues), whilst also interfering with host defense mechanisms [19].

Secreted proteins, which are actively released away from the bacterium, can harm the host by affecting host immunity [20]. SCIN (staphylococcal complement inhibitor, *scn*) and CHIPS (Chemotaxis inhibitory protein of *S. aureus*, *chp*) negatively affect the progress of complement by impeding the activity of complement-associated enzymes [21] or phagocyte recruitment, respectively [22,23]. Panton Valentine Leukocidin (*pvl*), a well-known toxin that targets immune cells directly, forms β-barrel pores, resulting in cellular lysis and the subsequent death of phagocytes [24].

Many *S. aureus* VFs are multifunctional and have additional—or often redundant—roles. For example, ClfA (clumping factor A) and SdrE (serine aspartate repeat protein E) belong to the MSCRAMM family of adhesins, yet also participate in immune evasion by binding host complement regulators to subvert complement-mediated opsonization on the *S. aureus* surface [25,26]. Both staphylococcal Protein A (*spA*) and staphylococcal binder of immunoglobulin (*sbi*) bind antibody by the Fc region [27] and can be surface-bound or secreted. Thus, functional redundancies and/or multipurpose VFs create challenges for elucidating the role of VFs and specific disease manifestations. However, in the context of toxin-related diseases, some causative genes have been identified, such as *eta/etb* (staphylococcal scalded skin syndrome [28]) and *tsst-1* (toxic shock syndrome) [29]. Further, some VFs are designated as risk factors for specific infections, such as *bbp* in osteomyelitis [30], and *pvl* in osteomyelitis, lung infection, and severe infections [30,31,32]. Evidence also suggests that the presence of *pvl* or *tsst-1* may indicate elevated antibiotic resistance [33,34]. Thus, determining VF-associated gene carriage in clinical isolates will shed light on the potential for *S. aureus* to cause disease.

As such, we sought to gain a better understanding of *S. aureus* virulence potential in isolates associated with communities of Southeastern Virginia. Using whole-genomic sequencing and targeted genomics, we screened 122 clinical isolates collected from patients of a children’s hospital in Norfolk, VA USA, to characterize lineage information (clonal complex and sequence type) as well as the carriage of a panel of clinically relevant VF-associated genes (Table 1). These data were analyzed against identified drug resistance/sensitivity profiles from isolate-specific antibiogram data. Infection type and methicillin resistance/sensitivity were used as additional discriminators to examine the relationship between lineage, VF gene carriage, and antibiotic resistance. Thus, this study provides information on VF prevalence and their association with infection type or antibiotic resistance and identifies common pathogenic determinants to support the development of targeted treatment strategies.

## 2. Materials and Methods

### 2.1. Bacteria

Community-associated *S. aureus* isolates were obtained as de-identified, discarded specimens from a children’s hospital in Norfolk, VA, and transferred in accordance with IRB 18-05-EX-0109; no human samples were used in this study. Isolates were identified as *S. aureus* via matrix-assisted laser desorption ionization time-of-flight (MALDI-TOF) mass spectrometry using the MALDI Biotyper Sirius CA System. Characterization of antibiotic resistance or sensitivity was determined using BD Phoenix PMIC 109 panel, a broth microdilution method utilizing cation-adjusted BD broth with 2-fold serial dilutions, and read every 20 min up to 16 h. Clinical and Laboratory Standards Institute and Antimicrobial Susceptibility Testing (CLSI AST) guideline rules were applied for validation, with six reference strains (*S. aureus* ATCC 29213, 25923, BAA-976 and BAA-977; *Enterococcus faecalis* ATCC 29212 and 51299) for quality control. See Table 2 for associated antibiotics and applied ranges. Isolates with demonstrated resistance or sensitivity to oxacillin were considered to be MRSA or MSSA, respectively. Infections of the blood or urine were deemed invasive due to infection location. SSTI infections were not classified as invasive or superficial.

### 2.2. DNA Extraction

Isolates were cultured on Columbia agar (BD Difco, Franklin Lakes, NJ, USA) with 2% NaCl. Bacteria were resuspended in sterile deionized water then heated at 99 °C for 10 min to lyse the cells. Lysates were subjected to phase extraction with phenol/chloroform/isoamyl alcohol, 25:24:1 (Sigma-Aldrich, St. Louis, MO, USA), followed by ethanol precipitation, and washed with 70% ethanol to purify gDNA. Concentration and purity of gDNA were assessed with a Nanodrop spectrophotometer or Qubit 4 fluorometer. gDNA samples were stored at −80 °C until use.

### 2.3. Whole-Genome Sequencing and Assembly

gDNA samples were prepared using the Illumina DNA LP (M) Tagmentation and Nextera DNA CD index kits, per manufacturer’s instructions. Prepared samples were subjected to whole-genome sequencing (WGS) using the Illumina iSeq 100 system, with paired-end read length of 150 bp and depth of 30× reads. Assembly of contigs was accomplished with the Assembly module of the Local Run Manager (Illumina, San Diego, CA, USA), which contains onboard algorithms for error correction and quality control of raw sequence reads. Genome annotation was performed using the Prokka prokaryotic genome annotation software (Version 1.14.6) [57], available via the Galaxy Project. Individual gene searches were conducted using the NIH NCBI database BLAST tool.

### 2.4. Sanger Sequencing and PCR

Clonal complex (CC) and sequence type (ST) were determined using WGS contigs screened via the PubMLST database [58]. For reads in dispute, Sanger sequencing was performed (EVMS Molecular Core Facility) as previously described [59]. MRSA isolates were further characterized for SCC*mec* type using multiplex PCR, as described by Zhang, et al. [60,61]. For unsuccessful WGS (*n* = 2, blood), gDNA was investigated for select VF presence by end-point PCR (Table 3).

### 2.5. Statistics

Statistical analyses were performed using R (Version 4.2.3) and the packages “readxl”, “dplyr”, “ggplot2” and “ggcorrplot” [69,70,71,72,73]. The associations between categorical data were evaluated using Fisher’s exact test with the addition of Mehta and Patel’s (1985) hybrid algorithm [74]. We evaluated the relationships between the antibiograms and the infection type or methicillin resistance using chi-squared tests. We ran appropriate nonparametric tests for the response variables that exhibit non-normal characteristics. All statistical tests of inferences were assessed at the 5% alpha level.

## 3. Results

### 3.1. Sample Pool Composition

**Figure 1:**
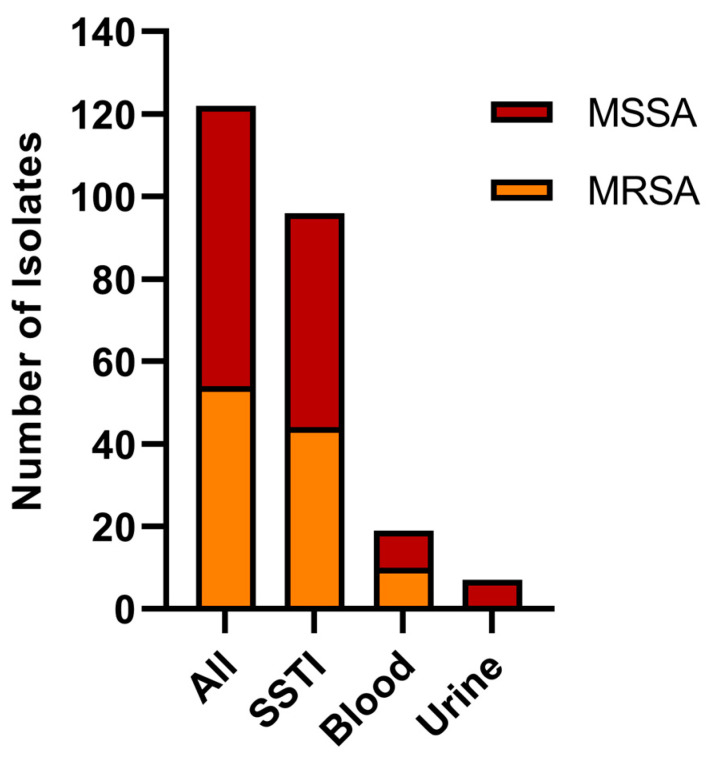
Isolate pool characterization of community association *S. aureus.* Isolate pool characterization of community associated *S. aureus*. MRSA and MSSA members are subdivided by infection type.

The isolate pool included in this study is depicted in Figure 1 and described in Table 4, categorized by infection type and methicillin resistance/sensitivity (*n* = 122). In total, 44% of sequenced isolates were MRSA (*n* = 54), and 56% were MSSA (*n* = 68), with most isolates from SSTI, followed by blood, then urinary tract infections. Two blood MRSA isolates were characterized using Sanger sequencing and end-point PCR for select VF genes, due to unsuccessful WGS runs.

### 3.2. Sequence Type, Clonal Complex, and SCCmec Distribution

To understand the lineage composition of the isolate pool (*n* = 122), we performed genomic analysis, revealing 31 unique sequence types (ST) within 9 recognized and 12 unknown/unrecognized clonal complexes (CCs). The most common lineage was CC 8 and ST 8; however, CC 8 isolates also represented the STs 72, 1159, 1181, 2176, and 7361 [58]. STs with low representation comprised 34 isolates, with 9 CA-MRSA and 25 CA-MSSA belonging to an additional 7 and 19 STs, respectively, and 3 additional CCs. See Supplementary Materials for a complete tally.

**Figure 2:**
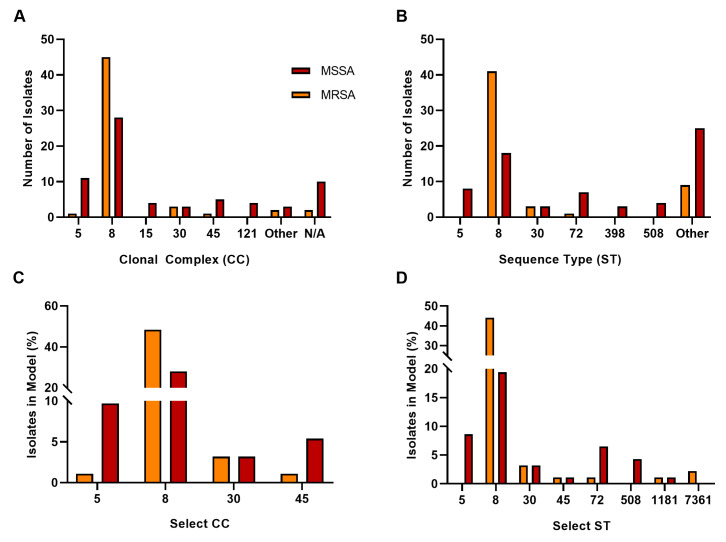
Isolate lineage analyses for MRSA vs. MSSA. Distribution of CC (**A**) and ST (**B**). Analysis of the most common CC (**C**) and corresponding ST (**D**). CCs and STs that contain ≥ 3 are shown. Groups not meeting this requirement were placed in “Other”. N/A represent members with recognized STs and no defined CC.

As the pool contained both MRSA and MSSA, we used methicillin resistance as a discriminator, indicating a significant relationship for CC and ST distribution (Figure 2A,B). A selection of four CCs and their corresponding STs (excluding urine isolates) were further analyzed to determine the direction of the significance (Figure 2C,D). Significance was retained for both CC and ST (*p* < 0.01).

**Figure 3:**
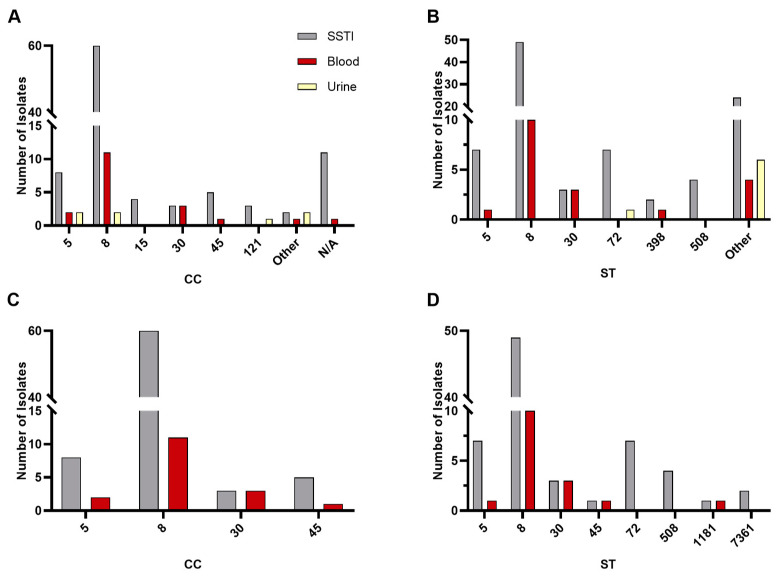
Isolate lineage analyses based on infection type. Distribution of CC (**A**) and ST (**B**). Represented CCs contain ≥ 4; represented STs contain ≥ 3. Groups not meeting this requirement were placed in “Other”. N/A represent members with recognized STs and no defined CC. Analysis of the four most common CC (**C**) and corresponding ST (**D**).

To determine whether infection type was a significant factor in lineage, we analyzed infection type with CC or ST. Whilst most isolates were from SSTI and belonged to CC 8 (as well as ST 8)—see Figure 3A,B—a significant relationship was found between infection type and CC or ST (*p* = 0.048, *p* < 0.01, respectively); however, the categories contributing to this test statistic are unknown. To further investigate this relationship, we examined the four most well represented CC and corresponding ST (excluding urine); see Figure 3C,D. However, no significant relationship was determined (*p* = 0.1214).

**Figure 4:**
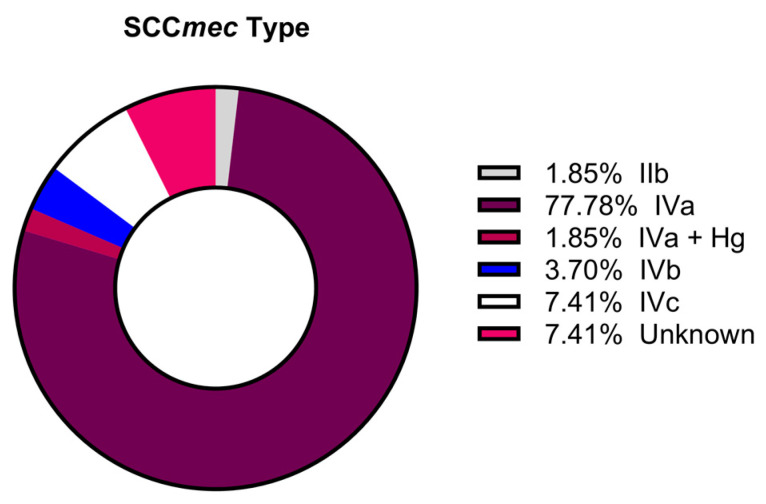
SCC*mec* distribution for MRSA.

As an additional feature relevant for characterizing the MRSA isolates, we examined the distribution of SCC*mec* (Figure 4), which demonstrated SCC*mec* type IVa to be dominant (77.8%). Four MRSA isolates were untypeable and may belong to an unknown type or those outside the tested type I–V distribution.

### 3.3. Antibiogram Data

**Figure 5:**
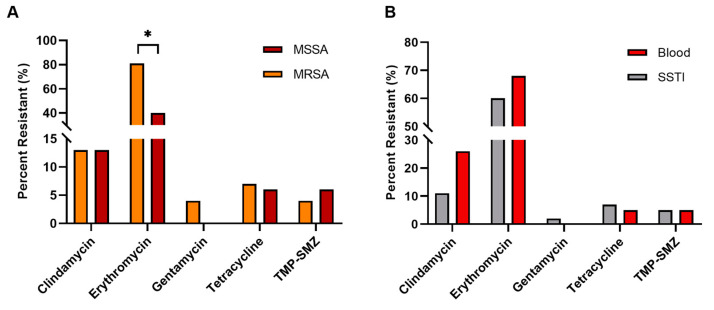
*S. aureus* antibiotic resistance. Antibiogram data for (**A**) MRSA vs. MSSA, (**B**) infection type. * *p* < 0.01. The following resistances are not depicted: rifampin (0%), vancomycin (0%), penicillin (99.2%), oxacillin (0% for MSSA, 100% for MRSA).

Antibiotic resistance is summarized in Figure 5. More than half of the pool (58%) were resistant to erythromycin (81% of MRSA, 40% of MSSA; *p* < 0.01, Χ^2^ = 16.797, df = 1); Figure 5A. Clindamycin resistance was equally distributed between MRSA and MSSA at 13%. Tetracycline resistance was present in 7% of isolates, and slightly more common in MRSA strains than MSSA (7% vs. 6%, respectively). Trimethoprim-sulfamethoxazole (TMP-SMZ) and gentamycin resistance were less common, with TMP-SMZ resistance evident in 5% (6% of MSSA, 4% of MRSA) and gentamycin resistance in 2% (MSSA). No significant difference in antibiotic resistance was determined based on infection type, blood vs. SSTI; however, clindamycin and erythromycin resistance were higher in isolates from blood infections vs. SSTI (Figure 5B). With the exception of penicillin, all urine samples were sensitive to the entire antibiotic panel. Excluding oxacillin and penicillin, MRSA isolates were found to have significantly higher average resistances (1.09 antibiotics/isolate) than the MSSA isolates (0.647 antibiotics/isolate) (Mann–Whitney U, W = 2449.5, *p* < 0.01). Analysis of the predominant CA-MRSA group (ST 8 with SCC*mec* IVa, *n* = 36), revealed no resistance to clindamycin (*p* = 0.02448, Χ^2^ = 5.0602, df = 1) compared to the entire CA-MRSA group (*n* = 54).

### 3.4. Virulence Factor Carriage

**Figure 6:**
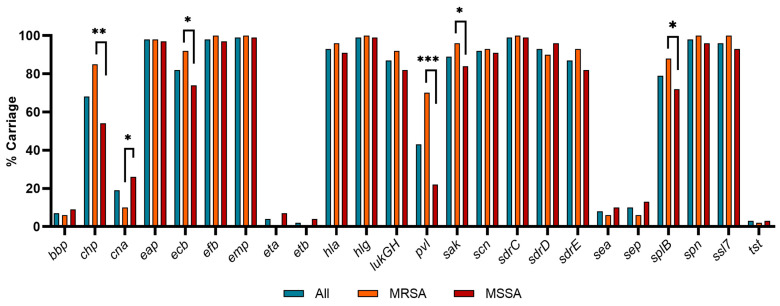
VF gene carriage based on methicillin resistance/sensitivity. For all isolates, MRSA, *n* = 52 (+2 for genes listed in Table 2) and MSSA groups (*n* = 68). *** *p* < 0.0001; ** *p* < 0.001, * *p* < 0.05.

As shown in Figure 6, most of the VF genes investigated were present in 50–80% of isolates, with higher frequency for MRSA strains in general. Some VFs, i.e., *eta* and *etb*, were only found in MSSA strains, whereas others (*bbp*, *cna*, *sea*, *sep*, *tst*), were less common (<10%) and not associated with methicillin resistance or sensitivity. *clfA*, *spA*, *sbi*, *scpA*, and *vwb* were present in all isolates and are described in Table 5. The carriage of six genes varied significantly based on methicillin resistance, according to Fisher’s exact test: *chp* (*p* = 0.004), *cna* (*p* = 0.0213), *ecb* (*p* = 0.009), *pvl* (*p* < 0.0001), *sak* (*p* = 0.0387), and *splB* (*p* = 0.0403); see Table 5. Whilst the direction of the significance could not be determined, their prevalence was proportionally higher for MRSA than MSSA for all but *cna*, where the trend was reversed.

**Figure 7:**
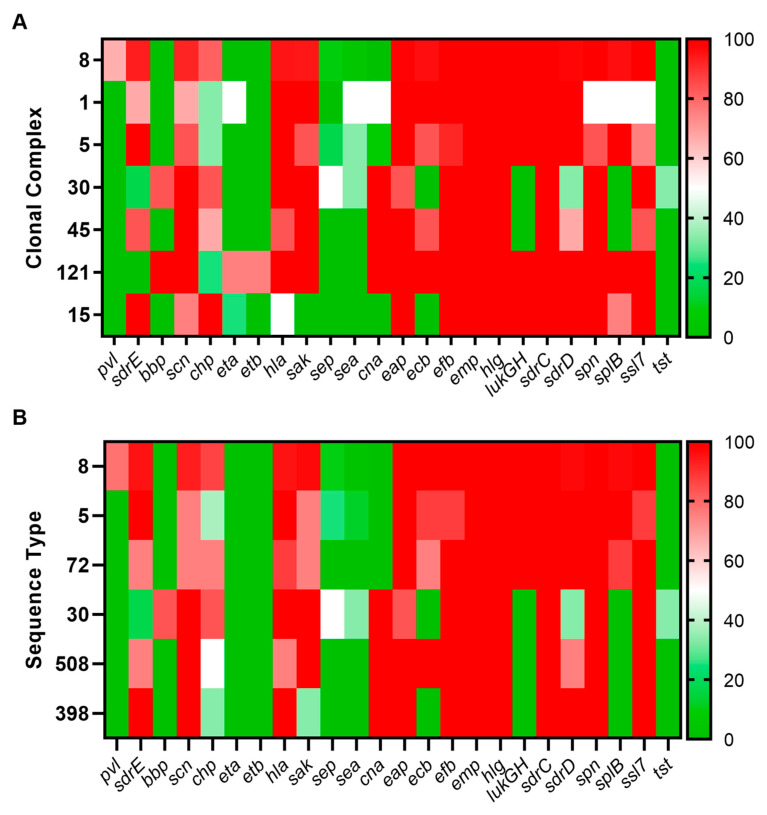
Heatmap of VF gene carriage based on lineage: (**A**) for CC containing at least 4 isolates; (**B**) for ST containing at least 3 isolates.

We also investigated whether any trends were evident for VF gene carriage based on lineage, excluding those identified as present in all isolates (Table 5). As shown in Figure 7, several genes are conserved across CC and ST for presence or absence as demonstrated by bold red (100% carriage) or bold green (no carriage).

**Figure 8:**
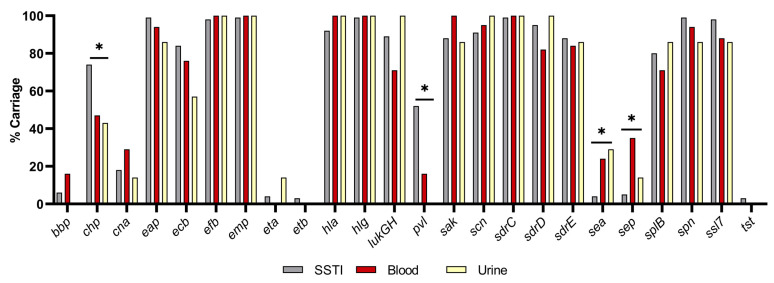
Virulence factor carriage by infection type. * *p* < 0.02.

In comparing VF carriage by infection type (Figure 8), four genes varied significantly *chp* (*p* = 0.0185), *pvl* (*p* < 0.01), *sea* (*p* < 0.01), and *sep* (*p* < 0.01), described in Table 6. A higher proportion of SSTI isolates carried *chp* and *pvl*, whilst *sep* carriage was highest in blood isolates. *sea* prevalence was similar for blood and urine isolates, with a much lower carriage in SSTI isolates.

**Figure 9:**
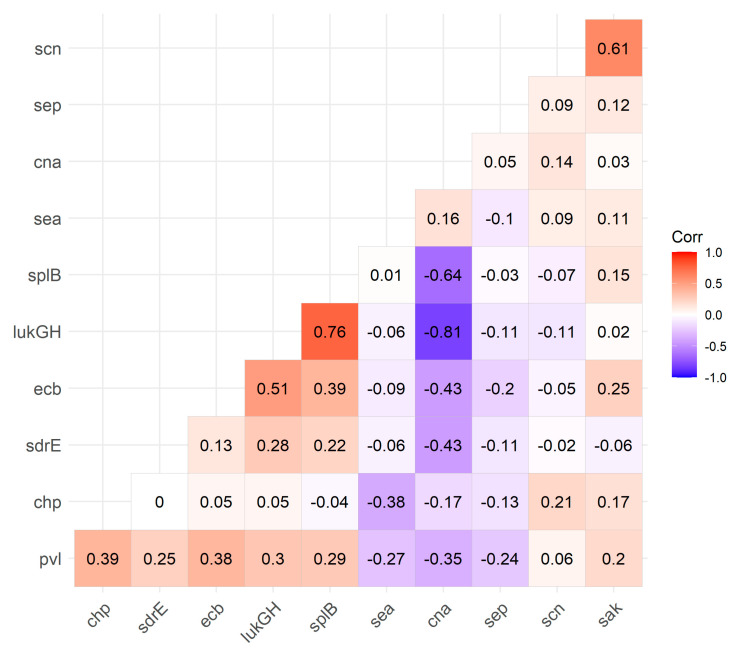
Correlation plot of the Pearson correlation coefficient for select VFs. *n* = 120 (+2 for genes listed in Table 3).

Using Pearson’s correlation, two pairs of genes showed a strong relationship and are associated with *lukGH*. A strong positive correlation was found between *lukGH* and *splB* (0.76), whilst a strong negative correlation was found between *lukGH* and *cna* (−0.81), as shown in Figure 9. All other correlations were moderate to low.

## 4. Discussion

### 4.1. Sequence Type, Clonal Complex, and SCCmec Distribution

Clonal analysis of *S. aureus* revealed important lineage information of the genomic makeup of the isolates found in communities of Southeastern Virginia. Although isolates were from a children’s hospital, pediatric trends reflect those of adults in the US, thus providing valuable data on *S. aureus* presence in this region [79]. CC 8, specifically ST 8, dominated for both MRSA and MSSA, as well as SSTI and blood isolates. Within the small set of urine-associated isolates, two were of CC 8, whereas none were ST 8, which may point to a clonal proclivity for a particular infection setting. A wider variation in ST was evident for the CA-MSSA isolates, which coincides with an expected greater diversity in this group, and perhaps a higher degree of unpredictability.

To further characterize the CA-MRSA isolates, we determined the type of SCC*mec*, a mobile genetic element that contains the *mecA* gene affording methicillin resistance [80]. In the CA-MRSA group, the most common SCC*mec* type was IVa. Of interest, USA300, the predominant CA-MRSA in the US, is ST 8 and contains SCC*mec* IVa, indicating that most of our CA-MRSA isolates may be USA300 clones. Although at low levels, additional SCC*mec* types were identified, which supports a varied presence in communities of Southeastern Virginia. USA300 has a well-documented history of infection across the US [81,82,83,84]. Given its success as a pathogen, having this lineage dominate our CA-MRSA pool is not surprising.

To investigate whether lineage correlates with infection, we cross-referenced the clonal data with infection type (SSTI, blood, and urine) revealing a significant difference for both CC and ST, thus identifying a connection between lineage and a particular disease state. Some CCs are reported to be highly associated with virulence models, such as infections of the blood or bone, indicating tropism for particular areas of the body [85,86]. Our analyses support these findings, providing additional sequence-type evidence to validate the usefulness of identifying lineage data.

### 4.2. Antibiograms

Community-associated *S. aureus* infections are commonly treated with dicloxacillin or cephalexin; however, patients allergic to penicillin may be prescribed erythromycin or similar antibiotics [87]. When MRSA is suspected or confirmed, sulfonamides or oxazolidinones are typically administered [87]. Serious infections are treated by considering documented sensitivities in the case of MRSA, or oxacillin for MSSA. Patients with penicillin allergy may be treated with clindamycin or vancomycin, but the former is not recommended when MRSA is suspected or confirmed [87]. Our data broadly support these guidelines. MRSA are known for frequent erythromycin resistance, but our finding of 40% erythromycin resistance in MSSA is still concerning and indicative of a potential change in the antibiotic resistance landscape [10]. The lack of rifampin or vancomycin resistance in our sample pool indicates that these antibiotics may remain viable treatment options for the near term. Clindamycin and erythromycin resistance were highest in blood isolates, indicating that this infection type may be at the highest risk of limited treatment options; this, coupled with the serious outcomes associated with *S. aureus* bacteremia, is of major concern. Whilst SSTI isolates exhibited a relatively low occurrence of clindamycin resistance, this trend may increase due to *S. aureus’* proclivity to developing resistance to antimicrobials. This is particularly worrisome for treating patients with drug allergies [11,14,15,87]. Interestingly, the predominant CA-MRSA lineage, ST 8 SCC*mec* IVa, exhibited no resistance to clindamycin. As USA300 isolates are resistant to fewer classes of antibiotics than their cohorts, this further supports that these isolates may be USA300 clones [83].

### 4.3. Virulence Factor Carriage

Five of the genes of interest were found in all clinical isolates screened: *clfA*, *scpA*, *sbi*, *spA*, and *vwb*. Whilst four of the five genes produce adhesins, all are involved in immune evasion, thus promoting *S. aureus* survival within the host, particularly where these properties intercept through the formation of biofilm [18]. As such, global carriage of these genes highlights their significance to *S. aureus* pathogenicity in multiple disease settings.

Six VFs were found to vary significantly with methicillin resistance: *chp*, *cna*, *ecb*, *pvl*, *sak*, and *splB*. Of these, only *cna* was more common in MSSA isolates than MRSA isolates. This falls in line with the literature, as MRSA are typically considered to be more virulent than MSSA. Again, these VFs are implicated in immune evasion, targeting the complement system or effector cells directly. As *chp* and *sak* belong to the same phage-encoded pathogenicity island, this suggests that their carriage would occur together [78]. *pvl* presence is implicated in severe disease and is associated with USA300. A high percentage of CA-MRSA in our sample pool were *pvl*+ (70%), indicating a high presence of this VF in MRSA isolates associated with communities of Southeastern Virginia.

Four VFs varied significantly with infection type: *chp*, *pvl*, *sea*, and *sep* (Figure 7 and Table 6). SSTI isolates were found to carry *chp* and *splB* more frequently, but were less likely to carry *sea*. Only *sep* was found most frequently in blood isolates, which supports a study by Calderwood, et al., demonstrating a significant link between *sep* carriage and the development of bacteremia [88]. Interestingly, the carriage of *chp* and *sea* was similar between blood and urine isolates, suggesting that their carriage may support invasive infections.

Several other VF genes were well represented across isolates and infection type, indicating their utility in *S. aureus* biology and corresponding pathogenicity. These included adhesins, toxins, proteases, and inhibitors, implicating a measurable benefit for their presence. A few of the VF genes were rare, namely the toxin genes *eta*, *etb*, and *tst*. Of interest, these were found in CA-MSSA only, and were predominantly of SSTI (no blood; one urine had *etb*). Some rare VFs were associated with a specific CC (e.g., *eta/etb* and CC 121), indicating that VF gene carriage may be predicted based on lineage, highlighting the benefit of isolate-specific data. CC 121 was uncommon in our sample pool (3%); however, this clone is globally disseminated and associated with the exfoliative toxins, with documented increasing resistance to vancomycin [89,90]. Although none of the isolates included in this study demonstrated resistance to vancomycin, the probability of reduced susceptibility to available antibiotics is likely to occur overtime, supporting the dire need to develop novel treatment strategies.

## 5. Conclusions

In this study, we determined the genetic variability of 122 *S. aureus* clinical isolates from patients of a pediatric hospital serving Southeastern Virginia. As pediatric trends reflect those of adults in the US, this study provides valuable data on *S. aureus* presence in this region [79]. Most of the isolates were associated with SSTI with significantly less from systemic blood and urinary tract infections, mirroring the dominant role of *S. aureus* as the largest single cause of SSTI worldwide. The most common lineage was CC 8, of which most were ST 8. In combination with SCC*mec* IVa dominating for CA-MRSA isolates, this lineage aligns with that of USA300. Additional STs identified for the CA-MSSA group indicates that CA-MSSA are likely to be less predictable than CA-MRSA due to an increased variability in lineage. Antibiogram data demonstrated a high prevalence of erythromycin resistance for isolates from both SSTI and blood infections, with blood isolates exhibiting the highest clindamycin resistance of any group analyzed. The carriage of select VF-associated genes varied significantly based on methicillin resistance and/or infection type; however, five genes were present in all isolates. The overwhelming commonality linking these genes is their association with immune evasion, in particular targeting the complement system and phagocytes. As *S. aureus* is often referred to as a master of immune evasion, these conserved genes, as well as those with high carriage, present an opportunity for further investigation. Understanding the level of VF gene carriage and pathogenic potential coupled with readily accessible clinical characteristics may support the development of better directed antistaphylococcal strategies.

## Figures and Tables

**Table 1:** Virulence factors of interest.

Gene	VF	Type ^1^	Group ^2^	Immune-Evasive	Ref.
*bbp*	Bone Sialoprotein Binding Protein	CW	MSCRAMM	Yes	[18]
*clfA*	Clumping Factor A	CW	MSCRAMM	Yes	[25,35]
*chp*	Chemotaxis Inhibitory Protein	S	Exoprotein	Yes	[22,23]
*cna*	Collagen Adhesin	CW	MSCRAMM	Yes	[36]
*eap*	Extracellular Adherence Protein	S	SERAM	Yes	[37]
*ecb*	Extracellular Complement Binding Protein	S	SERAM	Yes	[19,38,39]
*efb*	Extracellular Fibrinogen-Binding Protein	S	SERAM	Yes	[19,40,41]
*emp*	Extracellular Matrix Protein	S	SERAM	No	[42]
*eta*	Exfoliative Toxin A	S	Ex. Toxin	No	[28]
*etb*	Exfoliative Toxin B	S	Ex. Toxin	No	[28]
*hla*	α-hemolysin **or** Alpha Toxin	S	PF Toxin	Yes	[43]
*hlg*	γ-hemolysin **or** Gamma Toxin	S	PF Toxin	Yes	[44]
*lukAB/GH*	Leukocidin AB **or** Leukocidin GH	S	PF Toxin	Yes	[44]
*pvl*	Panton-Valentine Leukocidin	S	PF Toxin	Yes	[24]
*sak*	Staphylokinase	S	Protease	Yes	[45]
*sbi*	Staphylococcal Binder of Immunoglobulin	CW and S	Exoprotein	Yes	[27,41,46]
*scn*	Staphylococcal Complement Inhibitor	S	Exoprotein	Yes	[21]
*scpA*	Cysteine Protease Staphopain A	S	Protease	Yes	[47]
*sdrC*	Serine-Aspartate Repeat Protein C	CW	MSCRAMM	No	[48]
*sdrD*	Serine-Aspartate Repeat Protein D	CW	MSCRAMM	No	[49]
*sdrE*	Serine-Aspartate Repeat Protein E	CW	MSCRAMM	Yes	[18,26]
*sea*	Staphylococcal Enterotoxin A	S	Enterotoxin; Superantigen	Yes	[50]
*sep*	Staphylococcal Enterotoxin P	S	Enterotoxin	No	[51]
*spA*	Staphylococcal Protein A	CW and S	Exoprotein	Yes	[52]
*splB*	Serine Protease-Like Protein B	S	Protease	Yes	[53]
*spn*	Staphylococcal Peroxidase Inhibitor	S	Exoprotein	Yes	[54]
*ssl7*	Staphylococcal Superantigen-Like 7 Protein	S	Exoprotein	Yes	[55]
*tst*	Toxic Shock Syndrome Toxin	S	Superantigen	Yes	[29]
*vwb*	von Willebrand Factor-Binding Protein	S	SERAM	Yes	[19,56]

^1^ Type: secreted (S); cell-wall (CW). ^2^ Group: exfoliative toxin (Ex. Toxin); pore-forming toxin (PF Toxin).

**Table 2:** BD Phoenix panel.

		Infection Type *	
Antibiotic	Range (µg/mL)	Blood/SSTI	Urine
Clindamycin	0.5–2	X	N/A
Erythromycin	0.5–4	X	N/A
Gentamycin	1–8	X	X
Oxacillin	0.25–2	X	X
Penicillin G	0.125–8	X	X
Rifampin	0.5–2	X	X
Tetracycline	0.5–8	X	X
Trimethoprim- Sulfamethoxazole	0.5/9.5–2/38	X	X
Vancomycin	0.5–16	X	X
Nitrofurantoin	16–64	N/A	X

* Isolates associated with various infection type (blood, SSTI, or urine) were subjected to antibiotics as indicated by the X symbol. N/A indicates test not performed.

**Table 3:** PCR primers used for VF identification.

Gene	Forward Primer (5′-3′)	Reverse Primer (5′-3′)	Ref.
*bbp*	AACTACATCTAGTACTCAACAACAG	ATGTGCTTGAATAACACCATCATCT	[62]
*clfA*	ATTGGCGTGGCTTCAGTGCT	CGTTTCTTCCGTAGTTGCATTTG	[62]
*chp*	GGAATCAGTACACACCATCATTCAG	ATTTCTCAAACGTTCATCTAATTTTCC	[63]
*etb*	GTGGTAAAGGCGGACAACAT	TCAAATCGTTCCCCAAAGTG	[64]
*hla*	TATAGTCAGCTCAGTAACAACAACA	TGCATGCCATTTTCTTTATCATAAGTGAC	[63]
*pvl*	ATCATTAGGTAAAATGTCTGGACATGATCCA	GCATCAAGTGTATTGGATAGCAAAAGC	[65]
*scn*	GTTGATATTTTGCTTCTGACAT	AACGAAAAGTTAGCTAATGAAT	[66]
*sdrE*	AGAAAGTATACTGTAGGAACTG	GATGGTTTTGTAGTTACATCGT	[67]
*spA*	CAAACGGCACTACTGCTGAC	CATGGTTTGCTGGTTGCTTC	[68]

**Table 4:** Sample pool composition.

Category	*n*	Isolates
MRSA	54	44%
MSSA	68	56%
Blood	19	15.6%
SSTI	96	78.7%
Urine *	7	5.8%
Blood MRSA	10	8.2%
Blood MSSA	9	7.4%
SSTI MRSA	44	36%
SSTI MSSA	52	42.6%

*n* = 122; * All urine samples were CA-MSSA.

**Table 5:** Genes with 100% carriage.

Gene	VF	Description of Action	Ref.
*clfA*	Clumping Factor A	Binds human fibrinogen, involved in biofilm formation and *S. aureus*-mediated platelet aggregation. Contributes to immune evasion by binding complement regulator Factor I.	[25,35,75]
*scpA*	Cysteine Protease Staphopain A	Protease with inhibitory effects on complement pathways. Impairs phagocytosis by neutrophils.	[47]
*sbi*	Staphylococcal Binder of Immunoglobulin	Binds IgG Fc; binds and activates host plasminogen to intefere with complement-mediated opsonization.	[27,41]
*spA*	Staphylococcal Protein A	Binds IgG Fc and cross-links the Fab domain of IgM to subvert opsonization and phagocytosis.	[52]
*vwb*	von Willebrand Factor-Binding Protein	Secreted adhesin that binds to plasma components and induces blood clots. Assists in strengthening abscess walls.	[56]

**Table 6:** Genes with significant variation due to methicillin resistance or infection type.

Gene	VF	Description of Action	Carriage (%) *	Ref.
*chp*	Chemotaxis Inhibitory Protein	Inhibits fMLP- and C5a-induced chemotaxis of neutrophils and monocytes.	All: 68; 85 ^R^, 54 ^S^; 74 ^SSTI^, 47 ^B^, 43 ^U^	[22,23]
*cna*	Collagen Adhesin	Binds host collagen. Inhibits complement by binding the initiator protein C1q.	All: 19; 10 ^R^, 26^S^	[36,76]
*ecb*	Extra-cellular Complement Binding Protein	Impairs complement-mediated phagocytosis by binding complement C3b or C3, and reduces the cofactor activity of CR1.	All: 82; 92 ^R^, 74 ^S^	[38,39]
*pvl*	Panton-Valentine Leukocidin	Bi-component leukocidin that forms β-barrel pores in host cells, with high specificity to human neutrophils.	All: 43; 70 ^R^, 22 ^S^; 52 ^SSTI^, 16 ^B^, 0 ^U^	[24]
*sak*	Staphylokinase	Binds and activates host plasminogen to break down host extracellular matrices. Also removes IgG and C3b (opsonins) from the bacterial surface.	All: 89; 96 ^R^, 84 ^S^	[45]
*splB*	Serine Protease-Like Protein B	Cleaves and inactivates several complement components, inhibiting all three pathways, reducing bacterial killing via phagocytosis.	All: 79; 88 ^R^, 72 ^S^	[53]
*sea*	Staphylococcal Enterotoxin A	Commonly associated with food poisoning; causes emesis, diarrhea, and GI inflammation. Also known for nonspecific activation of T-cells, resulting in acute toxic shock.	All: 8; 4 ^SSTI^, 4 ^B^, 29 ^U^	[50,77]
*sep*	Staphylococcal Enterotoxin P	Related to and often on the same pathogenicity island as *sea*, though produces much milder symptoms.	All: 10; 5 ^SSTI^, 35 ^B^, 14 ^U^	[51,78]

* Superscript denotes the following: resistance type: ^R^ MRSA, ^S^ MSSA; infection type: ^SSTI^ SSTI, ^B^ blood, ^U^ urine.

## Data Availability

The data presented in this study are available in Supplementary Materials.

## Supplementary Materials

The following supporting information can be downloaded at: https://www.mdpi.com/article/10.3390/pathogens13010025/s1: gene carriage, antibiograms, CC group counts and ST group counts.
